# Cervical disc arthroplasty (CDA) versus anterior cervical discectomy and fusion (ACDF) in symptomatic cervical degenerative disc diseases (CDDDs): an updated meta-analysis of prospective randomized controlled trials (RCTs)

**DOI:** 10.1186/s40064-016-2851-8

**Published:** 2016-07-27

**Authors:** Lin Xie, Ming Liu, Fan Ding, Peng Li, Dezhang Ma

**Affiliations:** Department of Orthopedic Surgery, Wuhan Orthopedic Hospital, Wuhan Puai Hospital, Huazhong University of Science and Technology, Hanzheng Street 473#, Wuhan, 430033 Hubei Province China

**Keywords:** Cervical disc arthroplasty (CDA), Anterior cervical discectomy and fusion (ACDF), Cervical degenerative disc diseases (CDDDs), Meta-analysis, Randomized controlled trials (RCTs)

## Abstract

**Purpose:**

This meta-analysis of randomized controlled trials (RCTs) aims to evaluate the efficacy and safety in cervical disc arthroplasty (CDA) and anterior cervical discectomy and fusion (ACDF) for treating cervical degenerative disc diseases (CDDDs).

**Methods:**

The authors searched RCTs in the electronic databases (Cochrane Central Register of Controlled Trials, PubMed, EMBASE, Medline, Embase, Springer Link, Web of Knowledge, OVID and Google Scholar) from their establishment to march 2016 without language restrictions. We also manually searched the reference lists of articles and reviews for possible relevant studies. Researches on CDA versus ACDF in CDDDs were selected in this meta-analysis. The quality of all studies was assessed and effective data was pooled for this meta-analysis. Outcome measurements were surgical parameters (operative time, blood loss, and length of hospital stay), clinical indexes [neck disability index (NDI), neurological success, range of motion (ROM), Visual Analogue Score (VAS)], complications [the number of adverse events, adjacent segment disease (ASD), and reoperation]. Subgroup analysis, sensitivity analysis, and publication bias assessment were also performed, respectively. The meta-analysis was performed with software revman 5.3.

**Results:**

37 articles (20 RCTs) with a total 4004 patients (2212 in the CDA and 1792 in the ACDF) met inclusion criteria. Eight types of disc prostheses were used in the included studies. Patients were followed up for at least 2 years in all the studies. No statistically significant differences were found between CDA and ACDF for blood loss [SMD −0.02; 95 % CI (−0.20, 0.17)], length of hospital stay [MD −0.06; 95 % CI (−0.19, 0.06)]. Statistical differences were found between operative time [MD 14.22; 95 % CI (6.73, 21.71)], NDI [SMD −0.27; 95 % CI (−0.43, −0.10)], neurological success [RR 1.13; 95 % CI (1.08, 1.18)], ROM [MD 6.72; 95 % CI (5.72, 7.71)], VAS of neck [SMD −0.40; 95 % CI (−0.75, −0.04)], VAS of arm [SMD −0.55; 95 % CI (−1.04, −0.06)], the rate of adverse events [RR 0.72 95 % CI (0.53, 0.96)], the rate of ASD [RR 0.62; 95 % CI (0.43, 0.88)], and reoperation [RR 0.50; 95 % CI (0.39, 0.63)]. Subgroup analysis stratified by different types of disc prostheses was also performed.

**Conclusions:**

CDA is associated with higher clinical indexes and fewer complications than ACDF, indicating that it is a safe and effective treatment for CDDDs. However, the operative time of CDA is longer than ACDF. Because of some limitations, these findings should be interpreted with caution. Additional studies are needed. Large, definitive RCTs are needed.

## Background

Since anterior cervical discectomy and fusion (ACDF) was first described by Smith and Robinson, ACDF is widely accepted as a traditional gold standard surgical procedure for cervical degenerative disc diseases (CDDDs) which included radiculopathy and myelopathy (Bohlman et al. 1993). Clinical studies have reported good outcomes after ACDF (Yue et al. 2005). However, complications of ACDF such as dysphagia, dysphonia, loss of range of motion, pseud-arthrosis and adjacent segment degeneration (ASD) still confuse the spine surgeons.

To avoid complications after as ACDF, the cervical disc arthroplasty (CDA) is designed (DiAngelo et al. 2003). CDA is a treatment option for spine surgeons with the aim of preserving motion at the treated level. During the past decade, the CDA has emerged as an alternative treatment to ACDF and has been shown to provide the pain relief and functional improvements similar or superior to those of ACDF. However, complications of CDA such as instability and heterotopic ossification also confuse the spine surgeons (Zechmeister et al. 2011).

A few previous meta-analyses (Fallah et al. 2012; Gao et al. 2013, 2015; Jiang et al. 2012; Luo et al. 2015a, b; Li et al. 2015; Muheremu et al. 2015; Rao et al. 2015; Ren et al. 2014; Shriver et al. 2015; Verma et al. 2013; Wu et al. 2015; Xing et al. 2013; Yao et al. 2015; Yin et al. 2013; Yang et al. 2012; Yu et al. 2011; Zhu et al. 2016; Zhong et al. 2016) have focused on this problem, but they have different conclusions about whether CDA is superior to CDA in treating CDDDs (Table 1). They used single-site data which is part of a multicenter trial or missed some important data. In the same time, many randomized controlled trials (RCTs) comparing CDA with ACDF for the treatment of CDDDs were performed (Burkus et al. 2010, 2014; Cheng et al. 2009, 2011; Coric et al. 2011; Davis et al. 2013; Delamarter and Zigler 2013; Delamarter et al. 2010; Davis et al. 2015; Hisey et al. 2014, 2015; Heller et al. 2009; Kesman et al. 2012; Kelly et al. 2011; McAfee et al. 2010; Mummaneni et al. 2007; Murrey et al. 2008, 2009; Phillips et al. 2013, 2015; Nabhan et al. 2007a, b, c, 2011; Porchet and Metcalf 2004; Qizhi et al. 2014; Riina et al. 2008; Riew et al. 2008; Rozankovic et al. 2014; Sasso et al. 2007, 2008, 2011; Skeppholm et al. 2015; Vaccaro et al. 2013; Zhang et al. 2012, 2014; Zigler et al. 2013). Therefore, an updated meta-analysis is needed which is based on the latest high quality studies. To solve this problem, we performed an updated meta-analysis to compare the outcomes between CDA and ACDF in treating CDDDs.

**Table 1:**
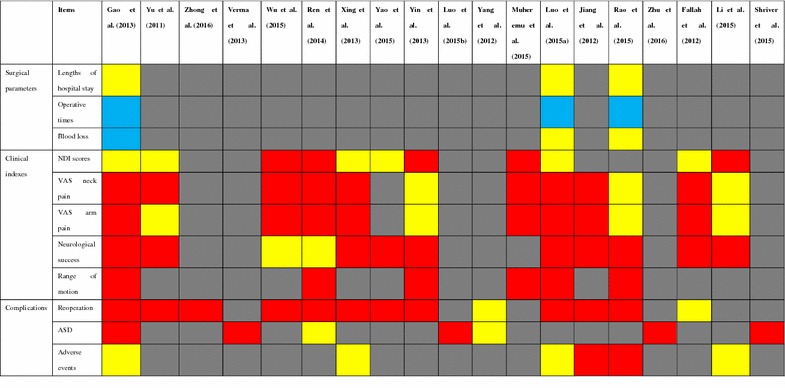
Results of previous meta-analysis

Red means favoring CDA; Yellow means no difference; Grey means not reporting; and blue means favoring ACDF

*NDI* neck disability index, *VAS* Visual Analogue Score, *ASD* adjacent segment disease

## Methods

### Search strategy

To make an exhaustive search of all relevant literatures, two independent reviewers (LX and ML) conducted a PRISMA (Preferred Reporting Items for Systematic Reviews and Meta-Analyses) guidelines. We searched RCTs in the online electronic databases (Cochrane Central Register of Controlled Trials, PubMed, EMBASE, Medline, Embase, Springer Link, Web of Knowledge, OVID and Google Scholar) from their establishment to march 2016 without language restrictions. We also manually searched the reference lists of articles and reviews for possible relevant studies. The following Mesh and free text search terms included: “anterior cervical decompression and fusion”, “anterior cervical discectomy and fusion”, “cervical disc replacement”, “disc prostheses” and “cervical arthroplasty” with a limit of “clinical trial”.

### Inclusion and exclusion criteria

Studies were eligible for inclusion if they met the following criteria: (1) RCTs comparing CDA with ACDF; (2) a minimum 2-year follow-up. Studies were excluded if they met the following criteria: nonrandomized studies, retrospective studies, reviews, commentaries, meta-analyses, and animal studies; duplicate publications of one trial; and single-site data as part of a multicenter trial. Two reviewers (LX and DZM) independently selected the potentially qualified trials according to the inclusion and exclusion criteria. Any disagreement was resolved by discussion and a conformity was reached.

### Data extraction

Study characteristics and secondary surgical outcomes were extracted independently by two reviewers (LX and ML) using a data extraction form, with discrepancies being arbitrated by consensus with a third reviewer (DZM). Informations extracted from studies included studies design, type of prostheses, center, numbers, age, the rate of male, the rate of follow up, surgical parameters (operative time, blood loss, and length of hospital stay), clinical indexes [neck disability index (NDI), neurological success, range of motion (ROM), Visual Analogue Score (VAS)], complications (the number of adverse events, ASD, and reoperation). The time point of clinical indexes and complications is 24 months after operation.

### Quality assessment

Quality evaluation of methodology of included studies was performed according to the Cochrane Collaboration’s tool for assessing risk of bias. Reviewers (PL and FD) independently determined random sequence generation, allocation concealment, blinding of participants and personnel, blinding of outcome data, selective outcome reporting, intend to treat analysis, group similarity at baseline and other sources of bias.

### Statistical analysis

All data were performed with Review Manager 5.3 software (The Nordic Cochrane Center, Cochrane Collaboration, Copenhagen, Denmark). The relative risk (RR) and its 95 % confidence interval (CI) were calculated for count data. Standardized mean difference (SMD) or mean difference (MD) and its 95 % CI were calculated for continuous outcomes. *P* < 0.05 was considered to be statistically significant. Heterogeneity was assessed using Chi squared and *I*-squared tests. Values of *I*^2^ greater than 50 % with *P* < 0.05 were considered to be substantial heterogeneity. Subgroup analyses were applied to identify the source of the heterogeneity and random model was applied when significant heterogeneity was observed among the included studies.

## Results

### Search results

**Fig. 1:**
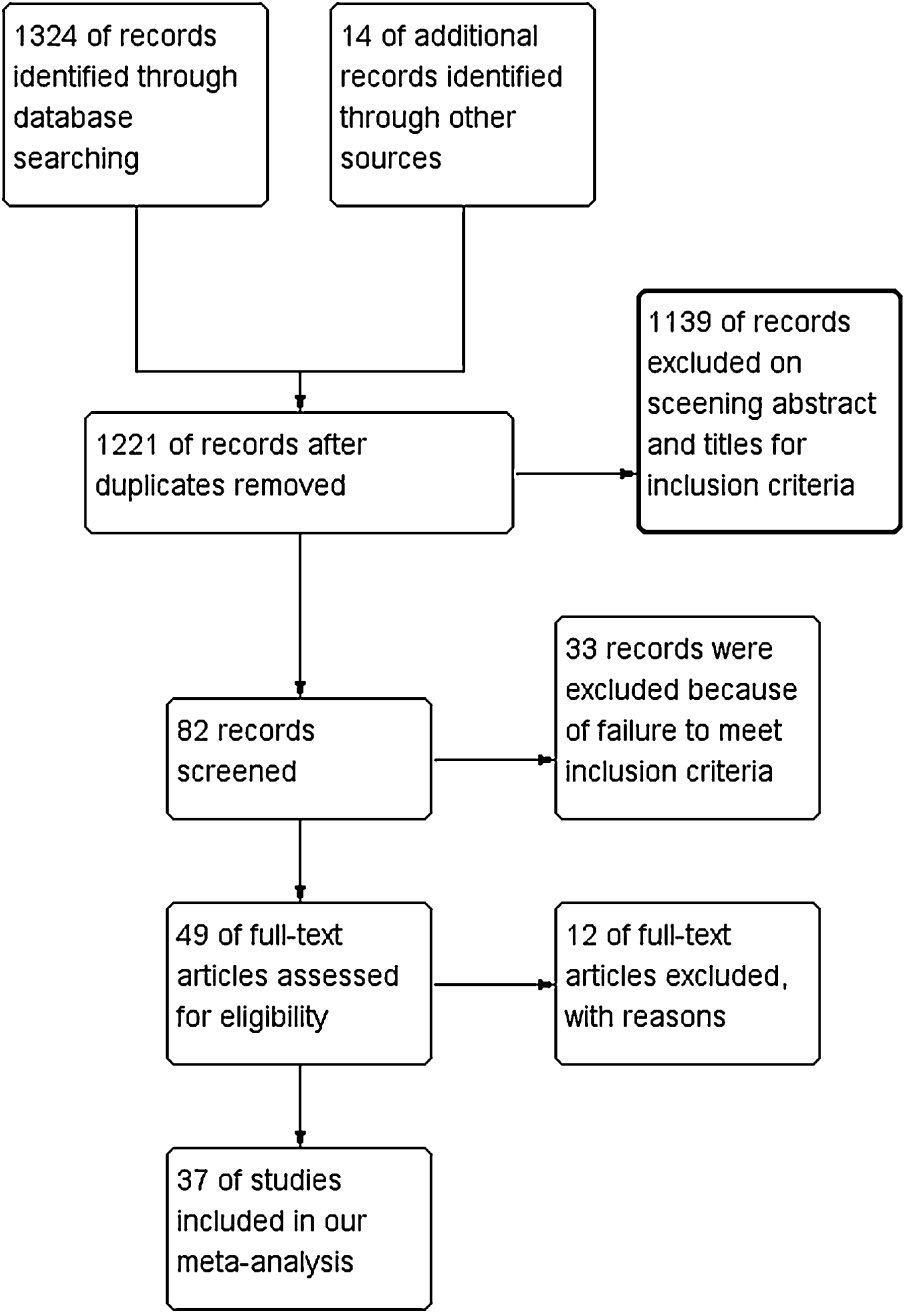
Study flow diagram

**Fig. 2:**
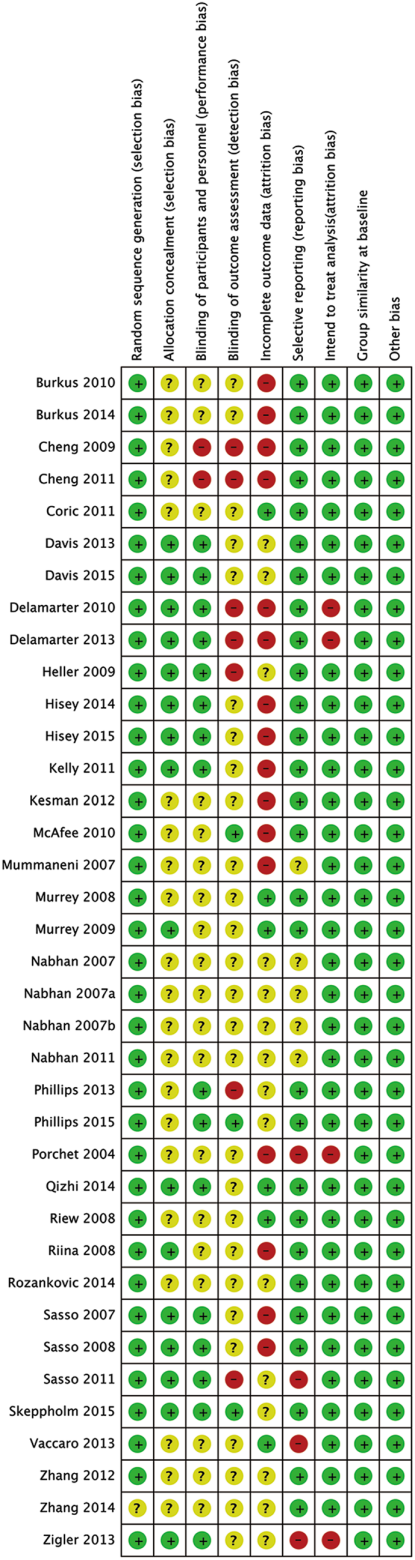
Assessment of risk of bias for the included studies is shown. + = low risk of bias; − = high risk of bias; ? = unclear risk of bias

 The details of the literature search and selection are displayed in Fig. 1. A total of 1338 relevant researches were identified through PubMed (N = 749), EMBASE (N = 389), CENTRAL (N = 128), and reference lists (N = 72). 1221 trials were remained after excluding the duplicates. After reviewing the titles and abstracts, 1184 trials were excluded because they did not reach the standard of inclusion criteria. A full text review was accessed in the retaining 37 studies, and finally, 20 RCTs with 4004 individuals (CDA = 2212, ACDF = 1792) were included in this meta-analysis (Burkus et al. 2010, 2014; Cheng et al. 2009, 2011; Coric et al. 2011; Davis et al. 2013; Delamarter and Zigler 2013; Delamarter et al. 2010; Davis et al. 2015; Hisey et al. 2014, 2015; Heller et al. 2009; Kesman et al. 2012; Kelly et al. 2011; McAfee et al. 2010; Mummaneni et al. 2007; Murrey et al. 2008, 2009; Phillips et al. 2013, 2015; Nabhan et al. 2007a, b, c, 2011; Porchet and Metcalf 2004; Qizhi et al. 2014; Riina et al. 2008; Riew et al. 2008; Rozankovic et al. 2014; Sasso et al. 2007, 2008, 2011; Skeppholm et al. 2015; Vaccaro et al. 2013; Zhang et al. 2012, 2014; Zigler et al. 2013) (Fig. 2). We recorded the characteristics of 37 included papers in Table 2 (Burkus et al. 2010, 2014; Cheng et al. 2009, 2011; Coric et al. 2011; Davis et al. 2013; Delamarter and Zigler 2013; Delamarter et al. 2010; Davis et al. 2015; Hisey et al. 2014, 2015; Heller et al. 2009; Kesman et al. 2012; Kelly et al. 2011; McAfee et al. 2010; Mummaneni et al. 2007; Murrey et al. 2008, 2009; Phillips et al. 2013; Nabhan et al. 2007a, b, c, 2011; Phillips et al. 2015; Porchet and Metcalf 2004; Qizhi et al. 2014; Riina et al. 2008; Riew et al. 2008; Rozankovic et al. 2014; Sasso et al. 2007, 2008, 2011; Skeppholm et al. 2015; Vaccaro et al. 2013; Zhang et al. 2012, 2014; Zigler et al. 2013).

**Table 2:** Summary of study characteristics

Studies	Design	Prostheses	Country	Center	No (CDA/ACDF)	Age(CDA/ACDF)	Male % (CDA/ACDF)	Follow up (%)
Zhang et al. (2012)	RCT	Bryan (Medtronic Sofamor Danek, Memphis, TN, USA)	China	Multicenter	120 60/60	44.8/45.6	58.3/53.3	NA
Sasso et al. (2007, 2008, 2011), Heller et al. (2009), Riew et al. (2008)	RCT	Bryan (Medtronic Sofamor Danek, Memphis, TN, USA)	USA	Multicenter	463 242/221	44.4/44.7	45.5/55.1	73
	RCT	Bryan (Medtronic Sofamor Danek, Memphis, TN, USA)	USA	Multicenter	199 106/93	44.5/44.4	17.0/16.1	NA
Cheng et al. (2009, 2011)	RCT	Bryan (Medtronic Sofamor Danek, Memphis, TN, USA)	USA	Singlecenter	83 41/42	47.2/47.7	51.2/54.8	98
Skeppholm et al. (2015)	RCT	Discover (DePuy Spine, Raynham, MA, USA)	Sweden	Multicenter	153 73/80	42.2/41.7	49.4//47.1	91
Rozankovic et al. (2014)	RCT	Discover (DePuy Spine, Raynham, MA, USA)	Croatia	Singlecenter	101 51/50	41/41	49.0/50.0	100
Qizhi et al. (2014)	RCT	Discover (DePuy Spine, Raynham, MA, USA)	China	Singlecenter	30 14/16	47/48	64.2/68.8	100
Coric et al. (2011)	RCT	Kineflex (Spinal Motion Inc, Mountain View, CA, USA)	USA	Multicenter	269 136/133	43.7/43.9	37.5/44/4	87
Zhang et al. (2014)	RCT	Mobi-C (LDR Medical, Troyes, France)	USA	Multicenter	111 55/56	44.8/46.7	45.5/46.4	NA
Hisey et al. (2014, 2015)	RCT	Mobi-C (LDR Medical, Troyes, France)	USA	Multicenter	245 164/81	43/44	47.6/44.4	75
Davis et al. (2013, 2015)	RCT	Mobi-C (LDR Medical, Troyes, France)	USA	Multicenter	330 225/105	45.3/46.2	50.2/42.9	86
Phillips et al. (2013, 2015)	RCT	PCM (NuVasive Inc, San Diego, CA, USA)	USA	Multicenter	293 163/130	45.3/43.7	52.8/51.9	72.70
McAfee et al. (2010)	RCT	PCM (NuVasive Inc, San Diego, CA, USA)	USA	Multicenter	251 151/100	45/44	50.3/47.0	NA
Riina et al. (2008)	RCT	Prestige ST (Medtronic Sofamor Danek, Memphis, TN, USA)	USA	Singlecenter	19 10.0/9.0	40.8/38.1	20.0/33.3	84.20
Riew et al. (2008)	RCT	Prestige ST (Medtronic Sofamor Danek, Memphis, TN, USA)	USA	Multicenter	111 59/52	43.4/46.0	49.2/40.4	NA
Porchet and Metcalf (2004)	RCT	Prestige II (Medtronic Sofamor Danek, Memphis, TN, USA)	Switzerland	Multicenter	55 27/28	44.3/43.2	63.0/40.4	67.3
Mummaneni et al. (2007), Burkus et al. (2010, 2014)	RCT	Prestige ST (Medtronic Sofamor Danek, Memphis, TN, USA)	USA	Multicenter	541 276/265	43.3/43/9	46.4/46	77.80
Nabhan et al. (2007a, b, c, 2011)	RCT	ProDisc-C (Synthes Inc, West Chester, PA,USA)	Germany	Singlecenter	41 20/21	44	23/18	NA
Murrey et al. (2008, 2009), Delamarter et al. (2010), Kelly et al. (2011), Kesman et al. (2012), Zigler et al. (2013), Delamarter and Zigler (2013)	RCT	ProDisc-C (Synthes Inc, West Chester, PA,USA)	USA	Multicenter	209 103/106	42.1/43/5	44.7/46.2	95.20
Vaccaro et al. (2013)	RCT	SECURE-C (Globus Medical, Audubon, PA, USA)	USA	Multicenter	380 236/144	43.4/44.4	53.6/48.6	87.10

*CDA* cervical disc arthroplasty, *ACDF* anterior cervical discectomy and fusion, *RCT* randomized controlled trial, *NA* no available

### Quality assessment

the risk of bias of each study was independently assessed by two authors (ML, LX), in accordance with the Cochrane risk of bias tool, which defines nine aspects: (1) random sequence generation (selection bias); (2) allocation concealment (selection bias); (3) blinding of participants (performance bias); (4) blinding of treatment providers (performance bias); (5) blinding of outcome assessors (detection bias); (6) intention to treat (attrition bias); (7) selective reporting (reporting bias); (8) comparable study groups; and (9) other bias. A qualification of risk of bias, including low risk, unclear risk, or high risk, was provided (Fig. 2). The final qualification for each study was determined by consensus among three authors (ML, LX, and DZM).

### Study characteristics

All 37 studies included in this meta-analysis were RCTs, 14 RCTs were conducted in the United States, and the other six were done in Asia and Europe. The years of publication ranged from 2004 to 2015. Sample sizes ranged from 19 to 582, and a total of 4004 patients (2212 in the CDA and 1792 in the ACDF) were enrolled in the 37 studies. Disc prostheses types included Bryan (Medtronic Sofamor Danek, Memphis, TN, USA), Discover (DePuy Spine, Raynham, MA, USA), Kineflex (Spinal Motion Inc, Mountain View, CA, USA), Mobi-C (LDR Medical, Troyes, France), PCM (NuVasive Inc, San Diego, CA, USA), Prestige ST (Medtronic Sofamor Danek, Memphis, TN, USA), ProDisc-C (Synthes Inc, West Chester, PA, USA), SECURE-C (Globus Medical, Audubon, PA, USA). Fifteen of the included studies were multi-center trials; Five were a single-center trials (Table 2).

### Outcome analysis of surgical parameters

**Fig. 3:**
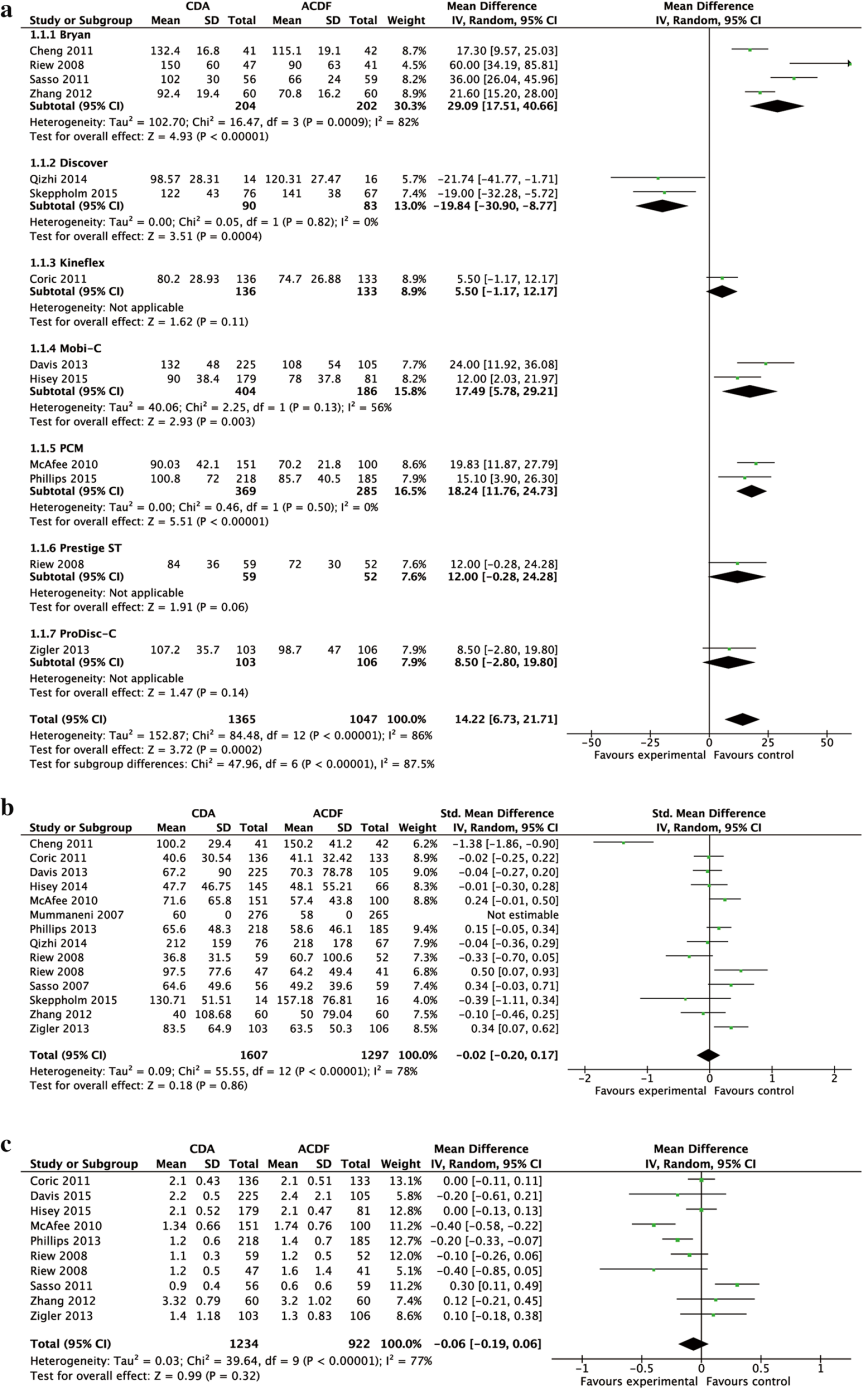
Forest plot for surgical parameters [operative time (**a**), blood loss (**b**), and length of hospital stay (**c**)]

The operation time of the CDA group was significantly longer than that of the ACDF group [MD 14.22; 95 % CI (6.73, 21.71)] (Fig. 3a). However, the amount of blood loss showed no significant difference between two groups [SMD −0.02; 95 % CI (−0.20, 0.17)] (Fig. 3b). Also, there was no significant difference in the length of hospital stay [MD −0.06; 95 % CI (−0.19, 0.06)] (Fig. 3c).

### Outcome analysis of clinical indexes

**Fig. 4:**
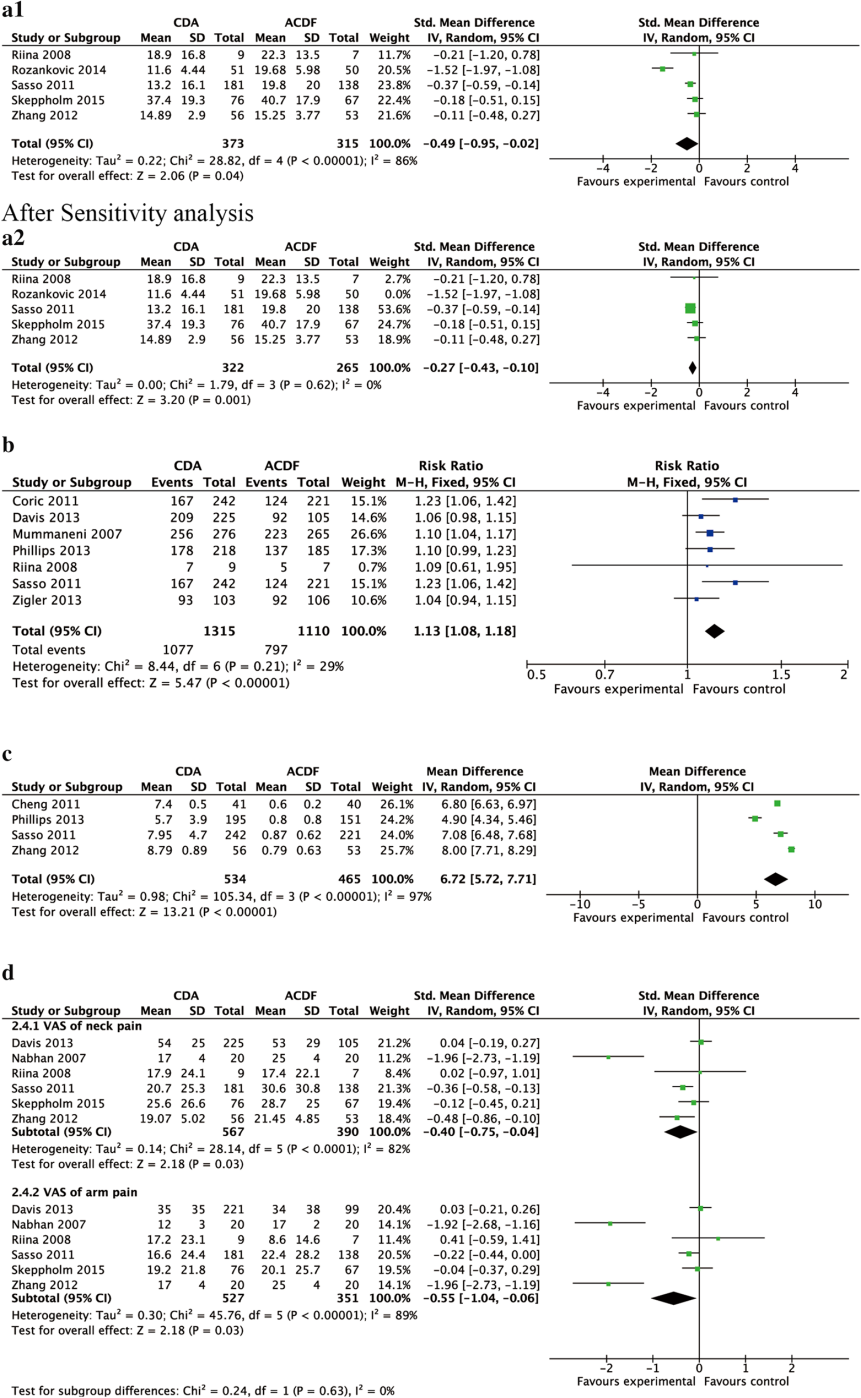
Forest plot for clinical indexes [neck disability index (NDI) (**a1**, **a2**), neurological success (**b**), range of motion (ROM) (**c**), Visual Analogue Score (VAS) (**d**)]

The NDI score [SMD −0.27; 95 % CI (−0.43, −0.10)] (Fig. 4a2), VAS of neck [SMD −0.40; 95 % CI (−0.75, −0.04)] (Fig. 4d) and VAS of arm [SMD −0.55; 95 % CI (−1.04, −0.06)] (Fig. 4d) of the CDA group was significantly lower than that of the ACDF group. The rate of neurological success [RR 1.13; 95 % CI (1.08, 1.18)] (Fig. 4b) and ROM [MD 6.72; 95 % CI (5.72, 7.71)] (Fig. 4c) was significantly higher than that of the ACDF group.

### Outcome analysis of complications

**Fig. 5:**
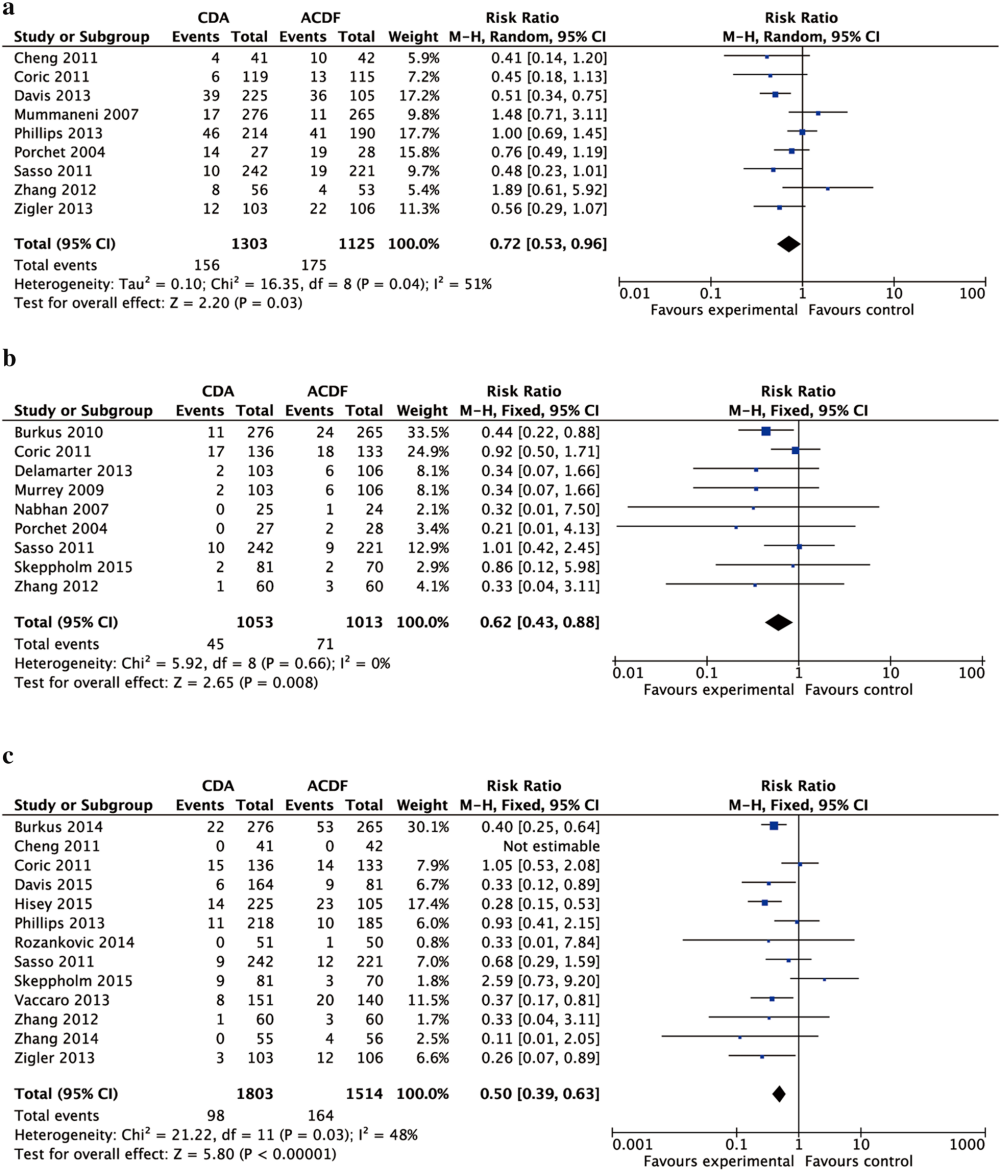
Forest plot for complications [the number of adverse events (**a**), adjacent segment disease (**b**), and reoperation (**c**)]

The rate of adverse events [RR 0.72 95 % CI (0.53, 0.96)], the rate of ASD [RR 0.62; 95 % CI (0.43, 0.88)], and reoperation [RR 0.50; 95 % CI (0.39, 0.63)] of the CDA group was significantly lower than that of the ACDF group (Fig. 5).

### Publication bias

The publication bias was evaluated by a funnel plot. The funnel plot shapes showed no obvious evidence of a symmetry. The results suggested that publication bias was not evident in this meta-analysis.

### Sensitivity analysis

Due to the high heterogeneity in the above analysis, we performed subgroup analysis in the meta-analysis based on different disc prostheses types. A sensitivity analysis was also conducted by removing one study at a time. We found that Rozankovic (Rozankovic et al. 2014) influenced the NDI scores in this analysis.

## Discussion

CDDDs can result in arm and neck pain, walking instability or a combination of symptoms which included myelopathy and radiculopathy. When symptoms do not respond to conservative treatment, operative treatment is considered. ACDF is an effective treatment for patients with symptomatic CDDDs (Bohlman et al. 1993). It has been performed for about 50 years. However, the loss of motion at the operated level can increase motion at the adjacent levels. ASD emerges gradually as a common complication. The original design purpose of CDA is to maintain the motion of operated level (DiAngelo et al. 2003). The technique is to restore and maintain the original biomechanics of cervical spine, which is attempted to prevent adjacent level degeneration at the operated segments. However, controversy still surrounds regarding whether CDA is better than ACDF.

There have been a few meta-analyses comparing the safety and efficacy between ACDF and CDA (Fallah et al. 2012; Gao et al. 2013, 2015; Jiang et al. 2012; Luo et al. 2015a, b; Li et al. 2015; Muheremu et al. 2015; Rao et al. 2015; Ren et al. 2014; Shriver et al. 2015; Verma et al. 2013; Wu et al. 2015; Xing et al. 2013; Yao et al. 2015; Yin et al. 2013; Yang et al. 2012; Yu et al. 2011; Zhu et al. 2016; Zhong et al. 2016). However, they have different conclusions (Table 1). To determine the effectiveness and safety of CDA for the treatment of symptomatic cervical disc disease, we performed a meta-analysis of RCTs on this subject. In our meta-analysis, we selected 20 RCTs comparing ACDF with CDA. We compared the surgical parameters (operative time, blood loss, and length of hospital stay), clinical indexes (NDI, neurological success, ROM, VAS), complications (the number of adverse events, ASD, and reoperation). The results of this meta-analysis indicated that CDA was superior to ACDF regarding fewer severe advents, fewer ASDs, fewer reoperations, better neurological success, greater ROM, lower NDI scores and greater neck and arm pain functional recovery. However, the outcomes of operative time are favor to the ACDF group.

Most of previous meta-analysis did not report the surgical parameters (Table 1). In our meta-analysis, the surgical parameters include operative time, blood loss and length of hospital stay. Our meta-analysis indicated that the operation time of the CDA group was significantly longer than that of the ACDF group. However, the amount of blood loss showed no significant difference between two groups. Also, there was no significant difference in the length of hospital stay. The operation time was associated with the different prosthesis types and the level of surgeons. Previous meta-analyses have different conclusions about the clinical indexes between CDA and ACDF (Table 1). In our meta-analysis, the clinical indexes include NDI, neurological success, ROM, VAS. Our study found that the CDA group had significantly better ROM and rate of neurological success, lower NDI scores, significantly lower neck pain scores, and lower arm pain scores than the ACDF group. The clinical indexes are associated with many factors such as decompression technique and ASD. Previous meta-analyses also have different conclusions about the complications between CDA and ACDF (Table 1). In our meta-analysis, the complications include Adverse events, ASD and reoperations. Our results indicated that adverse events, ASD and reoperations in ACDF group were more common than that in CDA group.

There are some strengths in our study. First, this is an updated meta-analysis with a larger sample size and included the latest RCTs to evaluate the efficacy and safety between CDA and ACDF in CDDDs. Second, we used Cochrane risk of bias to assess the quality of evidence.

Although this meta-analysis was performed with the best available evidence presently, some unavoidable weaknesses earned to be noted. First, the follow-up times of all the trials are different. In our paper, we choose 24 months as the time point of most trials, so we combined some articles. Second, many important studies only presented the VAS and NDI scores improvement (include the reductions and improvement) which was not the original data, so only 700–900 patients out of 4004 patients were available. Third, our results are affected by heterogeneity. For example, the results of operation time, blood loss, lengths of the hospital stay, ROM at the operated level, and VAS presented significant heterogeneity. Maybe various surgery interventions, different disc prostheses types and surgical technologies at different centers may influence the results. The results of this meta-analysis should be cautiously accepted. Large, definitive RCTs with longer-term follow-up are needed.

## Conclusions

In summary, our updated meta-analysis indicated the CDA was superior to ACDF regarding fewer severe advents, fewer ASDs, fewer reoperations, better neurological success, greater ROM and greater neck and arm pain functional recovery. However, the outcomes of operative time and NDI scores are favor to the ACDF group. More high-quality studies with longer term follow-up are needed to provide a better evaluation of the effectiveness and safety between the two treatments.

## Abbreviations

CDA: cervical disc arthroplasy
ACDF: anterior cervical discectomy and fusion
CDDDs: cervical degenerative disc diseases
RCTs: randomized controlled trials
MD: mean difference
NDI: neck disability index
ROM: range of motion
VAS: Visual Analogue Score
RR: relative risk
CI: confidence interval
